# Rocky Mountain Spotted Fever, Colombia

**DOI:** 10.3201/eid1307.060537

**Published:** 2007-07

**Authors:** Marylin Hidalgo, Leonora Orejuela, Patricia Fuya, Pilar Carrillo, Jorge Hernandez, Edgar Parra, Colette Keng, Melissa Small, Juan P. Olano, Donald Bouyer, Elizabeth Castaneda, David Walker, Gustavo Valbuena

**Affiliations:** * Instituto Nacional de Salud, Bogota, Colombia; † School of Medicine at Universidad de Los Andes, Bogota, Colombia; ‡ Secretaria de Salud de Cundinamarca, Colombia; § Hospital Salazar, Villeta, Cundinamarca, Colombia; ¶ University of Texas Medical Branch, Galveston, Texas, USA

**Keywords:** Rickettsia, Rocky Mountain spotted fever, Colombia, ticks, epidemiology, communicable diseases, emerging, dispatch

## Abstract

We investigated 2 fatal cases of Rocky Mountain spotted fever that occurred in 2003 and 2004 near the same locality in Colombia where the disease was first reported in the 1930s. A retrospective serosurvey of febrile patients showed that >21% of the serum samples had antibodies against spotted fever group rickettsiae.

Between July 1934 and August 1936, sixty-five cases of Rocky Mountain spotted fever (RMSF), 62 of them fatal, were reported from Tobia, Colombia (*1*). No reports of this disease (known locally as Fiebre de Tobia) have been produced from Colombia since, and currently RMSF is generally not included in the differential diagnoses of febrile syndromes.

We recently confirmed RMSF as the cause of death for 2 patients by PCR (*2*,*3*), sequencing, immunohistochemical tests (*4*), and culture (*5*) (Table 1). The first patient was a 32-year-old pregnant woman (26 weeks), who had abdominal pain, headache, and fever in December 2003; pharyngitis was diagnosed, and she received amoxicillin with no improvement. A cutaneous macular rash, hepatomegaly, hyperbilirubinemia, leukocytosis, and thrombocytopenia (50,000/μL) subsequently developed. She then experienced respiratory failure and died. One of her relatives, as well as 2 dogs, had died a few days earlier with similar symptoms. Another sick dog rapidly recovered after receiving doxycycline.

**Table 1:** Rocky Mountain spotted fever patients and findings, Colombia, 2003–2004

Patient	Confirmatory methods						Adult ticks collected in area where patients lived
	PCR		Sequence	IHA*	Animal inoculation	Culture	
1	Genes	Primers	Homology with *Rickettsia rickettsii*	Positive with rabbit anti–spotted fever group rickettsial antibody	Not done	Not done	15 *Amblyomma cajennense,* 184 *Rhipicephalus sanguineus,* 7 *Anocentor nitens,* and 8 *Amblyomma* spp.
	17-kDa	17kD1/2	100% (Sheila Smith)				
2	Genes	Primers	Homology with *R. rickettsii*	Positive with rabbit anti–spotted fever group rickettsial antibody	24 h and 48 h after fever onset, 2 guinea pigs were euthanized for culture and PCR analysis of spleens	Vero cells with cytopathic changes after 1 week	36 *A. cajennense,* 13 *R. sanguineus,* and 38 *Boophilus microplus*
	*gltA*	CS78/323	99% (Bitterroot and others)				
	*gltA*	CS5/6	94% (Bitterroot)				
	*OmpA*	190.70/701	98% (strain 1995HO2 and others)				
	*OmpB*	rOmpB.20-2788	98% (GenBank accession no. X16353.1)				

*Immunohistochemical analysis.

The second patient was a 31-year-old previously healthy man who went to the local hospital in May 2004 with fever and severe headache; dengue was diagnosed clinically. Three days later, he became stuporous and was admitted to the hospital. Within a short period, seizures developed and he became comatose. He died a few hours later.

The 2 patients lived near the towns of Villeta and Tobia, Cundinamarca, Colombia. The histopathologic findings of both patients were similar and consisted of vascular congestion; interstitial edema; frequent nonoccluding thrombi (mainly in the lungs); and multiple foci of perivascular lymphocytic and monocytic infiltration in all viscera, including the brain. The lungs showed marked interstitial inflammatory infiltrates. Immunohistochemical analysis showed rickettsiae in the microvascular endothelium of all studied organs, including brain, liver, spleen, and lungs of both patients (Figure).

**Figure:**
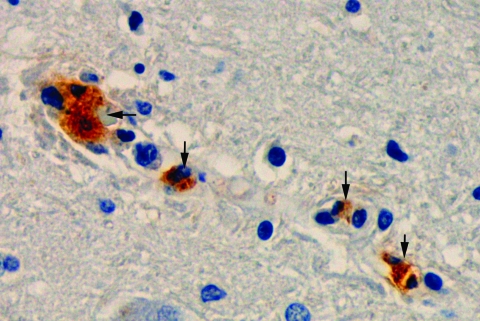
Immunohistochemical analysis shows the presence of spotted fever group rickettsiae (brown) in vessels of brain of a patient with fatal Rocky Mountain spotted fever (magnification ×400).

Several weeks after these events, we collected and identified adult male and female ticks from the farms and surroundings where the patients had lived (Table 1). We found ticks of the species *Amblyomma cajennense*, a known vector of spotted fever group rickettsioses in Latin America (*6*–*9*), and *Rhipicephalus sanguineus*, recently documented as a vector for *Rickettsia rickettsii* (*10*).

To begin to clarify the magnitude of spotted fever group rickettsioses as a public health problem in Colombia, we tested the following samples for spotted fever group rickettsiae by immunofluorescence assay (IFA) (*11*): 1) 64 serum samples from a national Colombian surveillance system (2001–2004) that studies malaria, dengue, and yellow fever (Instituto Nacional de Salud, Colombia); and 2) 96 serum samples from a regional (the state where the reported patients lived) surveillance system (2000–2001) for dengue (Secretaria de Salud de Cundinamarca, Colombia). Serum samples showing distinctly fluorescent rickettsiae at a >1:64 dilution were considered positive. We found immunoglobulin G (IgG) and IgM antibodies against spotted fever group rickettsiae (*R. rickettsii* was used as antigen) but not against typhus group rickettsiae (*R. typhi* was used as antigen) (Table 2). These data suggest that spotted fever group rickettsioses may be a frequent cause of febrile illnesses, not only in the state where the reported patients lived but also in various other regions of Colombia. Since there is strong cross-reactivity among rickettsial species when IFA is used as an antibody-detection technique, other spotted fever group rickettsiae, including those recently described in Latin America (*R. parkeri* and *R. felis*) could explain the assay results (*12*,*13*). Furthermore, most of these patients received a clinical diagnosis of dengue, an endemic disease in Colombia that appears to have become an umbrella diagnosis under which other diseases are assigned. A similar situation was recently described in Mexico (*14*).

**Table 2:** Titers of antibodies to spotted fever group rickettsiae (antigen: *Rickettsia rickettsii)* by indirect immunofluorescence antibody assay*

Surveillance program†	No. tested	IgG			IgM				States of origin
		No. (%) positive	Titer	n	No. (%) positive	Titer	n	No. also positive for IgG	
National	64	3 (4.7)	128	1	1 (1.5)	512	1	0	Santander, Guaviare, Caldas
			256	2					
Regional	96	21 (21.9)	64	3	20 (20.8)	64	3		Cundinamarca
			128	10		128	4		
			256	4		256	9	10	
			512	3		512	3		
			1,024	1		1,024	1		

*Ig, immunoglobulin. †Acute Febrile Disease Surveillance Programs.

RMSF in Colombia is seldom considered in the differential diagnosis for febrile disease; possible causes include the lack of an adequate diagnostic infrastructure and the invisibility of tick- and fleaborne infectious diseases in most medical curricula. The problem is further compounded by the presence of numerous agents (many transmitted by arthropod vectors) that produce nonspecific febrile syndromes during the early stages of the disease. Most of those agents are viruses that, unlike rickettsiae, have no specific treatment; thus, physicians might not feel compelled to use antimicrobial agents. Given the lack of appropriate and inexpensive diagnostic tests that are useful in the acute stage and that can be implemented in small rural hospitals, the best diagnostic tool available to healthcare personnel is clinical suspicion based on knowledge of the clinical manifestations (*15*), ecology, and epidemiology of rickettsioses. Physicians in areas where RMSF is endemic should consider prescribing a course of empirical treatment with doxycycline in patients who have high fever, severe headache, and myalgia, even in the absence of rash or history of tick bite, as both are frequently absent in RMSF. Such a treatment will not harm a patient with dengue or other viral infections and is likely to save the life of a patient infected with *R. rickettsii*.
